# Shape anisotropy revisited in single-digit nanometer magnetic tunnel junctions

**DOI:** 10.1038/s41467-018-03003-7

**Published:** 2018-02-14

**Authors:** K. Watanabe, B. Jinnai, S. Fukami, H. Sato, H. Ohno

**Affiliations:** 1 Laboratory for Nanoelectronics and Spintronics, Research Institute of Electrical Communication, Tohoku University, 2-1-1 Katahira, Aoba-ku, Sendai, 980-8577 Japan; 2 Center for Spintronics Integrated Systems, Tohoku University, 2-1-1 Katahira, Aoba-ku, Sendai, 980-8577 Japan; 3 Center for Spintronics Research Network, Tohoku University, 2-1-1 Katahira, Aoba-ku, Sendai, 980-8577 Japan; 4 Center for Innovative Integrated Electronic Systems, Tohoku University, 468-1 Aramaki Aza Aoba, Aoba-ku, Sendai, 980-0845 Japan; 5 WPI-Advanced Institute for Materials Research, Tohoku University, 2-1-1 Katahira, Aoba-ku, Sendai, 980-8577 Japan

## Abstract

Nanoscale magnetic tunnel junctions play a pivotal role in magnetoresistive random access memories. Successful implementation depends on a simultaneous achievement of low switching current for the magnetization switching by spin transfer torque and high thermal stability, along with a continuous reduction of junction size. Perpendicular easy-axis CoFeB/MgO stacks possessing interfacial anisotropy have paved the way down to 20-nm scale, below which a new approach needs to be explored. Here we show magnetic tunnel junctions that satisfy the requirements at ultrafine scale by revisiting shape anisotropy, which is a classical part of magnetic anisotropy but has not been fully utilized in the current perpendicular systems. Magnetization switching solely driven by current is achieved for junctions smaller than 10 nm where sufficient thermal stability is provided by shape anisotropy without adopting new material systems. This work is expected to push forward the development of magnetic tunnel junctions toward single-digit nm-scale nano-magnetics/spintronics.

## Abstract (web summary)

The thermal stability impedes the application of nanoscale magnetic tunnel junctions in electronic and spintronics devices. Here the authors achieved current-induced magnetization switching in magnetic tunnel junctions smaller than 10 nm with sufficient thermal stability due to the shape anisotropy without adding new material systems.

## Introduction

Since the theoretical prediction of spin transfer torque (STT)^1,2^ and early experimental demonstration of STT-induced magnetization switching^3–9^, its application to writing scheme in magnetoresistive random access memories (STT-MRAMs) has been a focus of spintronics research for the last two decades^10–19^. To make it a viable technology, the magnetic tunnel junctions (MTJs), the heart of the STT-MRAMs, is required to simultaneously show high tunnel magnetoresistance (TMR) ratio, low switching current (density), and high thermal stability factor *Δ* (≡ *E*/*k*_B_*T*, where *E* is the energy barrier between two possible states, *k*_B_ the Boltzmann constant, and *T* the absolute temperature). Furthermore, shrinking the size of the MTJ is necessary to achieve higher-density integration. The high TMR ratio was found to be attained by using MgO tunnel barrier^20,21^. Meanwhile, a lower switching current for a given *Δ* is known to be obtained in perpendicular easy-axis systems compared with in-plane ones^22–25^. However, this had posed a challenge for high-performance MTJs as most of the material systems with perpendicular anisotropy do not meet all the requirements simultaneously at a satisfactory level. This issue was settled by a discovery of perpendicular easy-axis CoFeB/MgO, which itself was a conventional in-plane system but with sufficient reduction of CoFeB thickness the buried interfacial perpendicular anisotropy^26,27^ emerged as a dominant factor to achieve a perpendicular easy axis^28–30^. A double CoFeB (or FeB)/MgO interface structure was subsequently introduced, which increases *Δ* by a factor of two^31^ due to an increased net anisotropy energy^31–33^, allowing scaling of MTJs down to around 20 nm^34^. Using the double-interface (Co)FeB-MgO MTJ, the STT-MRAMs are about to enter the market^17^. However, when one further reduces the MTJ size, the interfacial anisotropy approach reaches a physical limit in securing sufficient *Δ* while achieving STT-induced switching^35,36^. Thus, establishing a technology to pave the way toward sub-10 nm, or single-digit nm, MTJs is of pressing importance.

Here we show an unexplored approach to achieve perpendicular easy-axis MTJs meeting the requirements at ultrafine scales. We employ a commonly available material system FeB/MgO with the double-interface structure, but this time increase the thickness of FeB so that the shape anisotropy emerges as a dominant factor to keep the easy axis along the perpendicular direction. The fabricated MTJs exhibit a high *Δ* of more than 80, a sufficiently high value for most of the applications, and yet can be switched by STT at sizes smaller than 10 nm.

## Results

### Shape-anisotropy MTJs

We first describe the concept of the shape-anisotropy MTJ. The shape anisotropy energy originates from a classical magnetostatic interaction, and is defined as the energy difference between states for which magnetization is fully aligned along particular directions. In general, it acts to align the magnetization along the longest direction of the samples, that is, in-plane direction for membrane samples and longitudinal direction for needle-shaped samples. Assuming single-domain magnetization reversal, *Δ* of MTJ being subject to the shape, bulk, and interfacial anisotropies is expressed as

1 $${\mathit{\Delta}} \equiv \frac{E}{{k_{\mathrm{B}}T}} = \left( { - {\mathrm{{\delta} }}N\frac{{M_{\mathrm{S}}^2}}{{2\mu _0}}t + K_{\mathrm{b}}t + K_{\mathrm{i}}} \right)\frac{{\pi D^2}}{{4k_{\mathrm{B}}T}},$$

where *μ*_0_ is the permeability in free space, *K*_b_ and *K*_i_ are the bulk (magnetocrystalline) and interfacial-anisotropy energy densities, respectively, and *M*_S_, *t*, and *D* are the spontaneous magnetization, thickness, and diameter of the ferromagnetic layer, respectively. δ*N* is the difference in dimensionless demagnetization coefficient, or the shape anisotropy coefficient, between the perpendicular and in-plane directions, which is close to 1 when *D* ≫ *t*, as has been the case so far (see Supplementary Note 1 for more details). Next, the intrinsic critical current of STT switching *I*_C0_ is given as^1,2^

2 $$I_{{\mathrm{C}}0} = \alpha \frac{{2e\gamma }}{{\mu _{\mathrm{B}}g_{{\mathrm{STT}}}}}E,$$

where *α* is the Gilbert damping constant, *e* the elementary charge, *γ* the gyromagnetic ratio, *μ*_B_ the Bohr magneton, and *g*_STT_ the STT efficiency. Here we note that the intrinsic critical voltage *V*_C0_ is given by the product of the intrinsic critical current density *J*_C0_ = *I*_C0_/(π*D*^2^/4) and the resistance-area product *RA*, and should be small enough to secure reliability and integrated-circuit compatibility. Eq. (1) indicates that to obtain sufficient *Δ* (> 60–80^12,36^), one needs to employ material systems with high enough *K*_b_ or *K*_i_ and low enough *M*_S_ to overcome the negative shape anisotropy term. Meanwhile, Eq. (2) indicates that *I*_C0_ is proportional to a product of *α* and *E* and is not dependent on which term in the right-hand side of Eq. (1) dominates *E*. Thus, to achieve low *I*_C0_ for a given *Δ*, one needs to employ low *α* materials. The interfacial-anisotropy MTJ with double-interface structure^32^ satisfies the requirements on both *Δ* and *I*_C0_ down to around *D* = 20 nm^34^. However, further reduction of *D* inevitably degrades *Δ*, requiring a new approach. This leads us to revisit the shape anisotropy. If one can engineer δ*N* in Eq. (1), the perspective is expected to drastically change.

**Fig. 1:**
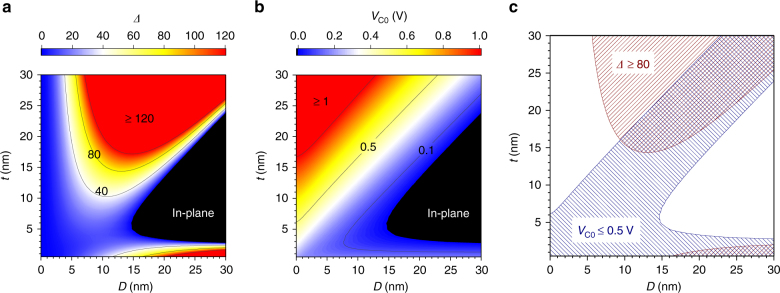
Calculation on MTJ performances. **a** Thermal stability factor *Δ* as a function of thickness *t* and diameter *D*. **b** Intrinsic critical voltage *V*_C0_ based on the Slonczewski model, where resistance-area product *RA* = 1 Ω μm^2^ is assumed. **c** Regions satisfying *Δ* ≥ 80 (red-hatched) and *V*_C0_ ≤ 0.5 V (blue-hatched). The regions in which the hatches are overlapped correspond to the design windows

Now, let us look at how *Δ* and *V*_C0_ vary with *D* and *t* through the change in δ*N*. Figure 1a, b show *D* and *t* dependence of *Δ* and *V*_C0_ calculated from Eqs. (1) and (2). Material parameters are set based on a typical double-interface MgO/FeB/MgO system (see Methods section). δ*N* is calculated analytically under several approximations (see Supplementary Note 1). The high-*Δ* region at bottom-right corner in Fig. 1a corresponds to the conventional interfacial-anisotropy MTJ. Here *Δ* ≥ 80 cannot be obtained when *D* ≲ 15 nm. However, when one further increases *t*, the easy axis turns to in-plane once and then becomes perpendicular again, entering a new region, that is, the shape-anisotropy MTJ. Here a region with sufficient *Δ* is seen even at 10 nm or less by virtue of the shape anisotropy. As for *V*_C0_ (Fig. 1b), it increases with *Δ* and 1/*D*^2^. Figure 1c shows design windows, in which necessary conditions of *Δ* ≥ 80 (red-hatched) and *V*_C0_ ≤ 0.5 V (blue-hatched) are satisfied, where *RA* = 1 Ω μm^2^ is assumed^37^. Shape-anisotropy approach offers a region satisfying the two requirements simultaneously at smaller *D* than for interfacial-anisotropy approach, holding promise for ultrafine STT-MRAMs.

### Characterization of blanket film

**Fig. 2:**
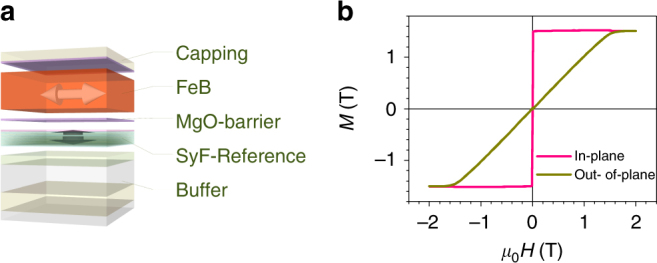
Stack structure and *M–H* curves. **a** Schematic of the blanket film of the stack structure, where the arrows show the expected magnetic easy axes. **b** Magnetization *M* in response to external magnetic field *H* applied along the in-plane and out-of-plane directions (*M*–*H* curves), for a blanket film comprising the recording layer part, MgO/FeB/MgO

Following the calculation results described above, we start the experimental parts from a blanket film study. On the basis of the calculation, we deposit 15-nm-thick ferromagnetic layer for the shape-anisotropy MTJ. The stack structure of MTJ is thermally oxidized Si substrate/Ta(5)/Ru(10)/Ta(15)/Pt(5)/[Co(0.4)/Pt(0.4)]_×6_/Co(0.4)/Ru(0.4)/Co(0.4)/[Pt(0.4)/Co(0.4)]_×2_/Ta(0.2)/CoFeB(1)/MgO(0.93)/FeB(15)/MgO(0.90)/Ru(5) (nominal thickness in nm), as depicted in Fig. 2a (see Methods section for details). FeB is employed as a material for the recording layer because it is expected to exhibit large *K*_i_ and small *α*. Note that large interfacial anisotropy helps obtaining perpendicular easy axis. Magnetization *M* responses to magnetic field *H* applied along the in-plane and out-of-plane directions (*M*–*H* curves) for a stack consisting of only the recording layer part (MgO/FeB/MgO) are shown in Fig. 2b. In-plane easy axis is confirmed from the two curves. The field to saturate *M* along the out-of-plane (hard axis) direction, is about 1.5 T, which is close to the saturated value of *M* (= 1.52 ± 0.01 T), indicating that the shape anisotropy is dominant in laying magnetization in the plane. *K*_i_ and *K*_b_ are evaluated to be 2.2 ± 0.1 mJ m^−2^ and (− 1.10 ± 0.07) × 10^5^ J m^−^^3^ from the thickness dependence of *M*–*H* curves. Ferromagnetic resonance (FMR) reveals that *α* of the recording layer is as small as 0.00425 ± 0.00003 (see Supplementary Note 2).

### Characterization of nano MTJs

**Fig. 3:**
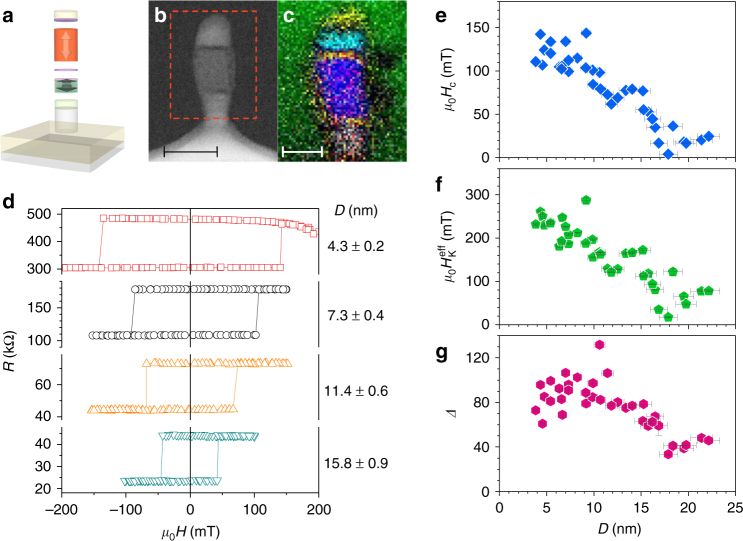
Magnetic field characterization of nano MTJs. **a** Schematic image of the nano MTJ, where the arrows show the expected magnetic easy axis. **b** Cross-sectional high-angle annular dark-field scanning transmission electron microscopy images of a MTJ after the ion milling process. Broken rectangle indicates the area for element mapping shown in **c**. The scale bar corresponds to 20 nm. **c** Corresponding image of element mapping using electron energy-loss spectroscopy. Representations of each color are B: red, N: green, O: yellow, Fe: dark blue, Co: white, Ru: light blue. The scale bar corresponds to 10 nm. **d** MTJ resistance *R* in response to perpendicular field *H* (*R*–*H* curves) for MTJs with various diameters *D*. **e**–**g** MTJ properties as a function of *D*. **e** Coercive field *H*_C_, **f** effective magnetic anisotropy field *H*_K_^eff^ and **g** thermal stability factor *Δ*. Error bars represent s.e.m, where those along the horizontal direction originate from uncertainty in determination of resistance-area product and those along the vertical direction originate from the fitting

Now we move onto the study of nano MTJs. Figure 3a shows a schematic illustration of the processed MTJ (see Methods section for the detail of integration process), and Figs. 3b, c, respectively, show the corresponding cross-sectional high-angle annular dark-field scanning transmission electron microscopy image and element mapping using electron energy-loss spectroscopy of a patterned MTJ. They reveal that *D* and *t* of the recording layer consisting of Fe and B are about 10 and 15 nm, respectively, as designed, within the region of perpendicular easy axis with high *Δ* (Fig. 1a).

Figure 3d shows the MTJ resistance *R* as a function of the out-of-plane field (*R*–*H* curves) for junctions with various *D*, where *D* of each MTJ is electrically determined from its *R* and *RA* (= 4.5 ± 0.5 Ω μm^2^) (see Supplementary Note 3 for detail). Square hysteresis loops are observed, indicating perpendicular easy axis owing to the shape anisotropy. Coercive field *H*_C_ increases as *D* decreases (Fig. 3e), also providing evidence that the shape anisotropy governs the stability of magnetization along the perpendicular direction. Next, we evaluate the effective anisotropy field *H*_K_^eff^ and *Δ* from switching probability using pulse magnetic field with 1 s duration (see Supplementary Note 4). Figures 3f, g show the obtained *H*_K_^eff^ and *Δ* as a function of *D* for a number of MTJs. *H*_K_^eff^ increases as *D* decreases, consistent with the trend of *H*_C_. Importantly, a sufficiently high *Δ* of more than 80 is obtained for MTJs with <10 nm in diameter and the values are consistent with our analytical calculation considering the shape anisotropy (Fig. 1a). For the smallest MTJ we measure (*D* = 3.8 ± 0.2 nm), *μ*_0_*H*_C_ = 111 mT, *μ*_0_*H*_K_^eff^ = 232 ± 3 mT, and *Δ* = 73 ± 1 are obtained. Deviation of observed trend between *Δ* and *D* from that expected from calculation, particularly a larger *Δ* and its smaller decreasing rate with *D* than that expected for the range of *D* less than around 15 nm, will be discussed later.

### Current-induced magnetization switching

**Fig. 4:**
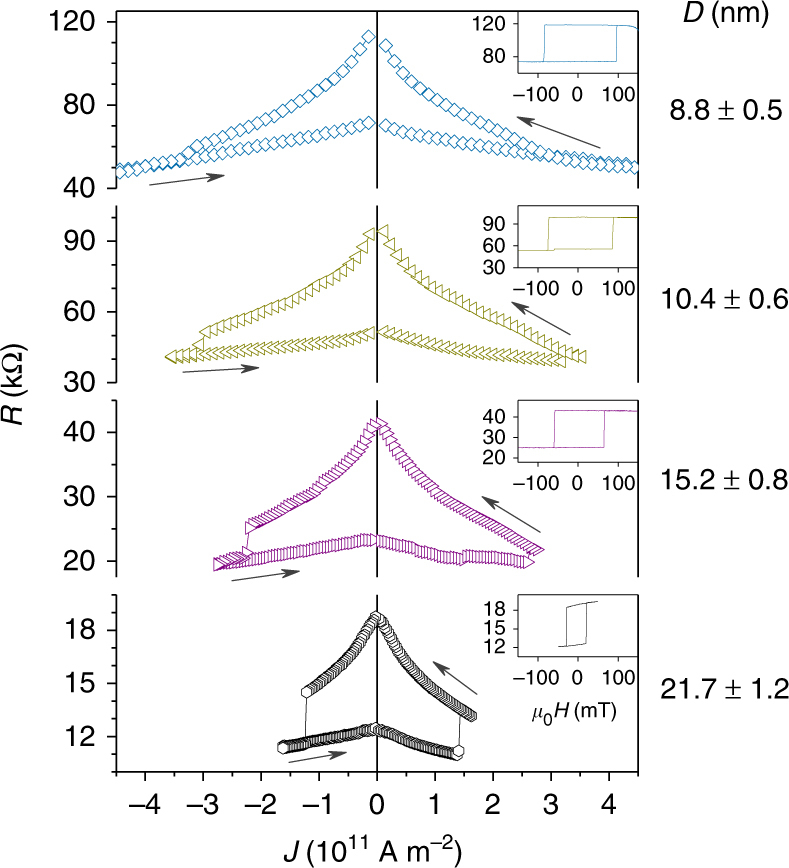
Current-induced switching properties. *R* in response to applied current density *J* (*R*–*J* curves) for MTJs with various diameters *D*. The insets show the *R*–*H* loop for the same MTJ

Finally, we show the switching of the MTJs with small *D* and high *Δ* using STT. Figure 4 shows typical *R* versus applied current density *J* (*R*–*J*) curves at zero magnetic fields. The pulse duration *τ*_p_ is 10 ms and *R* is measured during the pulse application. Inset shows the *R*–*H* curves for the same MTJ. Bidirectional magnetization switching is observed for each MTJ. In line with Eq. (2), the switching current density *J*_SW_ increases as *D* decreases due to an increase in *E*/*D*^2^. The smallest MTJ in which the switching is observed is 8.8 ± 0.5 nm in diameter. STT-induced switching for even smaller MTJs is expected to be achieved by reducing *t* and/or *RA*. From the observed *J*_SW_, *J*_C0_ can be calculated by^38^

3 $$J_{{\mathrm{C}}0} = \frac{{J_{{\mathrm{SW}}}}}{{1 - \frac{1}{{\mathit{\Delta}} }{\mathrm{ln}}\left( {\frac{{\tau _{\mathrm{p}}}}{{\tau _0}}} \right)}},$$

where *τ*_0_ is the inverse of attempt frequency (= 1 ns). For the case with 10.4-nm MTJ, where *J*_SW_ ≈ 3.2 × 10^11^ A m^−2^ and *Δ* ≈ 90, *J*_C0_ is derived to be 3.9 × 10^11^ A m^−2^, corresponding to the intrinsic critical current *I*_C0_ of 42 μA. This leads to the switching efficiency, defined as *Δ*/*I*_C0_, of 2.1 μA^−1^. This efficiency is almost the same as that in previously reported interfacial-anisotropy MTJs with similar size, which exhibited a much reduced *Δ*^30,34^. Meanwhile, the theoretical value calculated from Eq. (2) is 4.4 × 10^11^ A m^−2^. Good correspondence indicates that the observed current-induced switching is well described by the single-domain model considering the shape anisotropy, despite a large thickness of the recording layer.

## Discussion

As presented above, high-*Δ* (≥ 80) MTJs that can be switched by current are obtained at around and below 10 nm. The obtained *Δ* and *J*_SW_ are explained by an analytical model (Eqs. (1) and (2)), which considers the shape anisotropy. Here we discuss several other factors that may be necessary to be included to further describe the experiment. At first, while the values of material parameters such as *M*_S_, *K*_i_, *K*_b_, and *α* used for the calculation are determined based on blanket film studies, they could be different between the blanket film and nano MTJs; moreover they could vary with *D*. For instance, our recording layer is surrounded by a Si-N passivation layer, which could absorb B in the FeB recording layer and also could azotize the FeB recording layer. Such chemical reactions may result in variation of magnetic parameters. Second, the approximation used to calculate δ*N* (see Supplementary Note 1) may result in a certain deviation. Third, as can be seen in *R*–*J* loops shown in Fig. 4, TMR ratio has a bias-voltage dependence, resulting in overestimation of *g*_STT_ in Eq. (2). Finally, incoherent magnetization behavior and spin relaxation along the perpendicular direction may arise due to the thick recording layer, and there could be an upper limit on the thickness in which the present model holds true. Thus, to better understand the shape-anisotropy MTJs, the issues raised above have to be elucidated in future.

*J*_SW_, which is observed to be of the order of 10^11^ A m^−2^, has to be reduced for practical use in terms of drivability of cell transistors, active power consumption, reliability issues, and so on. This is particularly important when one considers further scaling of MTJ beyond 8.8 nm, since the theoretical model (Eq. (2)) predicts further increase in *J*_SW_ with decreasing *D* if *Δ* is kept at a certain level. The reduction of *J*_SW_ is expected to be achieved by adopting such as dual-reference layer structure^39,40^ and ultra-low-damping materials^41^. Because the shape-anisotropy MTJ can be formed with various structures using various material systems, there should remain a plenty of room of engineering to obtain better performance.

In summary, this study revisits the shape anisotropy, which has existed in conventional perpendicular MTJs but has so far counteracted the total perpendicular anisotropy because of its negative sign. We show that increasing the thickness of the recording layer makes the contribution positive and allows sufficient *Δ* of more than 80 at the 10-nm scale, which is not readily achieved by the conventional interfacial-anisotropy approach, as well as magnetization switching solely driven by current. The shape-anisotropy MTJ does not require any special materials, allowing us to choose from a broad range of material systems appropriate for the STT switching, which include large *M*_S_ materials. Moreover, the concept can be extended to ultrafine MTJs with in-plane easy axes, which may be suitable for spin-orbit torque-induced switching devices^42^. The present results provide an additional insight for the miniaturization of MTJs and open up the next era of nano-magnetics/spintronics.

## Methods

### Parameters for calculation

The analytical calculation of *Δ* and *J*_C0_ shown in Fig. 1 were conducted under an assumption of typical double-interface MgO/FeB/MgO structures. The used parameters were as follows: *M*_S_ = 1.5 T, *K*_i_ = 2.0 mJ m^−^^2^, *K*_b_ = 0 J m^−3^, *α* = 0.005 and TMR ratio = 100%. *g*_STT_ was deduced from the relative angle of magnetizations across the MgO barrier layer and spin polarization via TMR according to the Jullière model^43^.

### Film deposition

The stacks were deposited by magnetron sputtering at room temperature onto 3-inch thermally oxidized Si wafers. The stack structure was, from the substrate side, Ta(5)/Ru(10)/Ta(15)/Pt(5)/[Co(0.4)/Pt(0.4)]_×6_/Co(0.4)/Ru(0.4)/Co(0.4)/[Pt(0.4)/Co(0.4)]_×2_/Ta(0.2)/CoFeB(1)/MgO(0.93)/FeB(15)/MgO(0.90)/Ru(5) (nominal thickness in nm), where MgO layers were deposited by RF magnetron sputtering and the other layers were deposited by DC magnetron sputtering. The nominal composition of CoFeB was (Co_0.25_Fe_0.75_)_75_B_25_, while that in FeB was Fe_75_B_25_ (at. %). The reference layer has a synthetic ferrimagnetic (SyF) structure, to minimize the shift of *R*–*H* curves. For the blanket film studies, where magnetic parameters were quantified, stacks with the recording layer part, namely, Ta(5)/CoFeB(0.5)/MgO(1.2)/FeB(*t* = 2–30)/MgO(1.2)/CoFeB(0.5)/Ta(1)/Ru(5), were used.

### Nano-fabrication of MTJ

The deposited film was processed into nano MTJs with electrodes. After the film deposition we deposited 80-nm Ta by sputtering in situ and 50-nm Si-N by chemical vapor deposition ex situ. Then we patterned the MTJs using conventional electron beam lithography. A Si-N hard mask was formed using the resist pattern, followed by a formation of Ta hard mask using the Si-N hard mask through a reactive ion etching. MTJs were defined by multistep Ar ion milling using the Ta hard mask while varying the beam angle. During the milling, we monitored the secondary ion mass spectra and stopped the milling when the signal from the 10-nm-thick Ru layer appeared. The bottom Ta(5)/Ru(10) layers were used as an electrode for the electrical measurement. After the milling, the MTJ was ex situ covered with a Si-N passivation layer using chemical vapor deposition. This process might cause a formation of electrically dead regions through a side-wall oxidation (see Supplementary Note 3). After that, the wafer was covered with a spin-on glass and then etched-back with a reactive ion etching until the tops of the Ta hard mask were appeared. To form electrical contacts, Cr(5)/Au(100) was deposited on top of the MTJs and Ta/Ru underlayer using photolithography and lift-off. The processed wafers were post-annealed in vacuum for 1 h at 300 °C.

### Measurements

All the measurements were performed at room temperature. Vibrating sample magnetometer and ferromagnetic resonance using a TE_011_ microwave cavity were used for the blanket film study, and a standard electrical probing system, capable of applying out-of-plane or in-plane dc magnetic fields, was used for measurement on nano MTJs. A current source was used to supply current pulses through the MTJ, and a voltmeter was used to measure the dc voltage across the MTJ. A DC current with typical magnitude of 0.5 or 1 μA was supplied when measuring the MTJ resistance.

### Data availability

The data which support the findings of this work are available from the corresponding author upon reasonable request.

## Electronic supplementary material

Supplementary Information
