# Supplementary material for: Shape anisotropy revisited in single-digit nanometer magnetic tunnel junctions
Source: Nat Commun. 2018 Feb 14;9:663. doi: 10.1038/s41467-018-03003-7 (PMC5813113; doi:10.1038/s41467-018-03003-7)
Supplement: Supplementary file 1 — Supplementary Information [file 41467_2018_3003_MOESM1_ESM.pdf]

Shape anisotropy revisited in single-digit nanometer magnetic tunnel junctions

Watanabe *et al.*

Supplementary Note 1. Shape anisotropy coefficient δ*N*

The demagnetization coefficient is the ratio of the magnetostatic energy increase when the magnet considered is uniformly magnetized along arbitrary directions. In cartesian coordinate system, the coefficients N_x, N_y and N_z satisfy

$$N_x + N_y + N_z = 1. \tag{1}$$

When z axis is along the film normal direction and the magnet is isotropic in the film plane, *i.e.*, circular shape, x and y axes are equivalent and thus

$$N_x = N_y. \tag{2}$$

Since the magnetic anisotropy is defined as the difference in energy density between the states where magnetization aligns in the perpendicular and in-plane direction, shape anisotropy coefficient δN is given by

$$\delta N = N_z - N_x. \tag{3}$$

To analytically calculate the demagnetization coefficients of the recording layer with a cylindrical shape, we approximate it by a spheroidal single-domain nanomagnet with an aspect ratio $q = t/D$. Then, the demagnetization coefficient is known to be given by the following equations for two cases:

- Oblate spheroid ($q < 1$)[1],

$$N_x = \frac{q^2}{2(1-q^2)}\left(\frac{1}{q\sqrt{1-q^2}}\cos^{-1} q - 1\right). \tag{4}$$

- Prolate spheroid ($q \geq 1$)[2],

$$N_z = \frac{1}{q^2}\left\{\frac{1}{3} + \sum_{k=1}^{\infty} \frac{1}{2k+3}\left(1 - \frac{1}{q^2}\right)^k\right\}. \tag{5}$$

The calculated δN from Supplementary Equations 3, 4, and 5 is shown in Supplementary Figure 1a. The two curves with different approximations are connected smoothly with each other. Thus, we use Supplementary Equation 4 for $q \leq 0.9$ and Supplementary Equation 5 for $q > 0.9$ to calculate δN in equation (1) of the main body. Supplementary Figure 1b shows t and D

dependence of δN calculated from Supplementary Figure 1a. One can see that increasing t with D is required to satisfy the condition δN > 1, resulting in a requirement of increasing t with D for larger D region in Fig. 1a. On the other hand, for smaller D region, increasing t with decreasing D is necessary to keep the volume of the free layer.

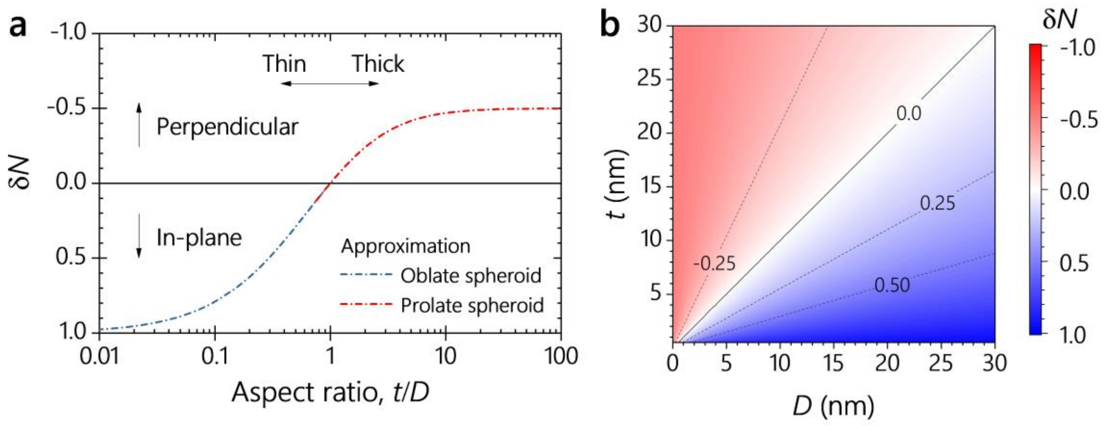

Supplementary Figure 1 | Calculated shape anisotropy coefficient δ*N*. a, Aspect ratio t/D dependence of δN calculated according to the two approximations[1,2]. **b**, δN as functions of t and D.

Supplementary Note 2. Magnetic properties of MgO/FeB/MgO blanket films

The magnetic properties of MgO/FeB/MgO stacks are evaluated with the vibrating sample magnetometer (VSM) and ferromagnetic resonance (FMR) measurements for blanket films. Supplementary Figures 2a and 2b, respectively, are the areal magnetic moment m in response to in-plane and out-of-plane magnetic field H (m-H curves). Saturated value of m (m_S) and areal effective magnetic anisotropy energy density $K_{eff}t$, obtained from the difference in the in-plane and out-of-plane m-H curves, are plotted as a function of FeB thickness t in Supplementary Figures 2c and 2d. We determine the spontaneous magnetization M_S, magnetic dead layer thickness t_d, interfacial and bulk magnetic anisotropy energy density K_i and K_b by the same way as used in Supplementary Reference 3. The obtained values are M_S = 1.52 ± 0.01 T, t_d = 0.0 ± 0.1 nm, K_i = 2.2 ± 0.1 mJ m^{-2}, K_b = (– 1.10 ± 0.07) ×10^5 J m^{-3}.

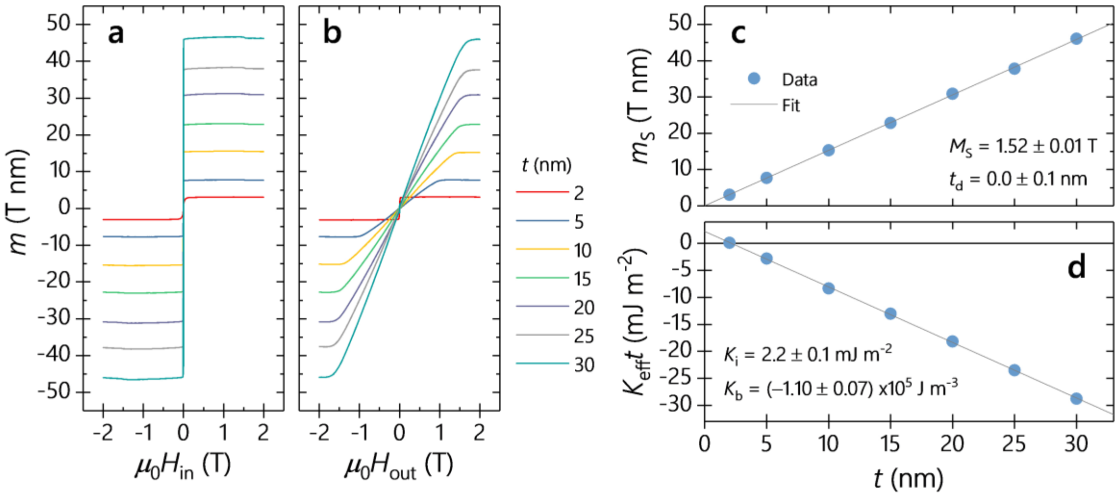

Supplementary Figure 2 | Magnetic properties characterization by VSM. a,b, Areal magnetic moment *m* versus applied magnetic field *H* (*m*-*H*) curves with various FeB thickness *t*. **a**, *m*-*H* curves along in-plane direction. **b**, *m*-*H* curves along out-of-plane direction. **c,d**, *t* dependence of **c**, m_S and **d**, $K_{eff}t$ as a function of *t* with linear fitting.

Supplementary Figure 3a shows the FMR spectra measured at various magnetic field angles θ_H from the film normal direction on a blanket film with t = 20 nm. Resonance field H_R and full-width at half-maximum ΔH of each spectrum is extracted and plotted in Supplementary Figures 3b and 3c, respectively, as a function of θ_H. The dependence of ΔH on θ_H is fitted with the same procedure including extrinsic effects such as two-magnon scattering and motional narrowing as that in Supplementary Reference 4. α is determined to be 0.00425 ± 0.00003.

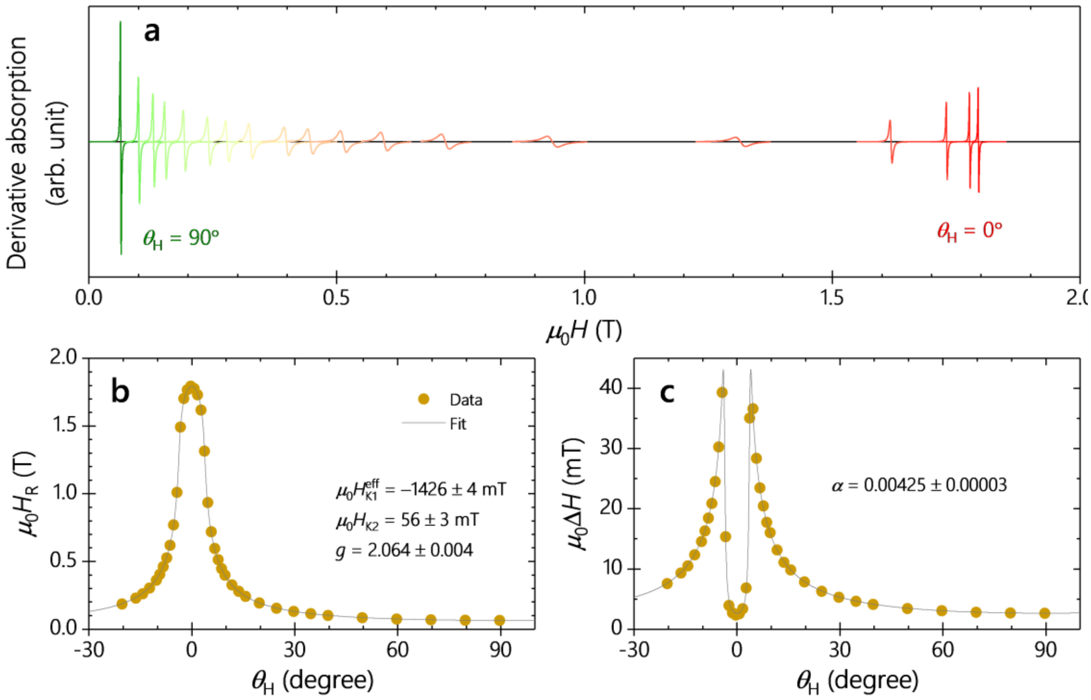

Supplementary Figure 3 | Magnetic property characterization by FMR measurement. **a**, FMR spectra measured at various magnetic field angles θ_H. **b**, θ_H dependence of resonance field H_R and its fitting. **c**, θ_H dependence of full-width at half-maximum ΔH and its fitting.

Supplementary Note 3. Resistance-area product *RA*

The square root of the electrically-measured values of conductance *G* at low resistive state of MTJs are plotted as a function of the physical diameter D_{TEM} of MgO barrier layer observed by transmission electron microscopy in Supplementary Figure 4. The resistance-area product *RA* is determined by a linear fitting according to Supplementary Equation 6, in which an electrically dead region[5] with a length of D_0 that surrounds electrically active region with diameter *D* is considered as a horizontal intercept:

$$G = \frac{1}{RA}\frac{\pi(D_{\mathrm{TEM}} - D_0)^2}{4}. \qquad (6)$$

RA of our MTJs is determined from the fitting to be 4.5 ± 0.5 Ω μm^2, with the depth of electrically dead region (D_0/2) to be 2.5 ± 0.7 nm.

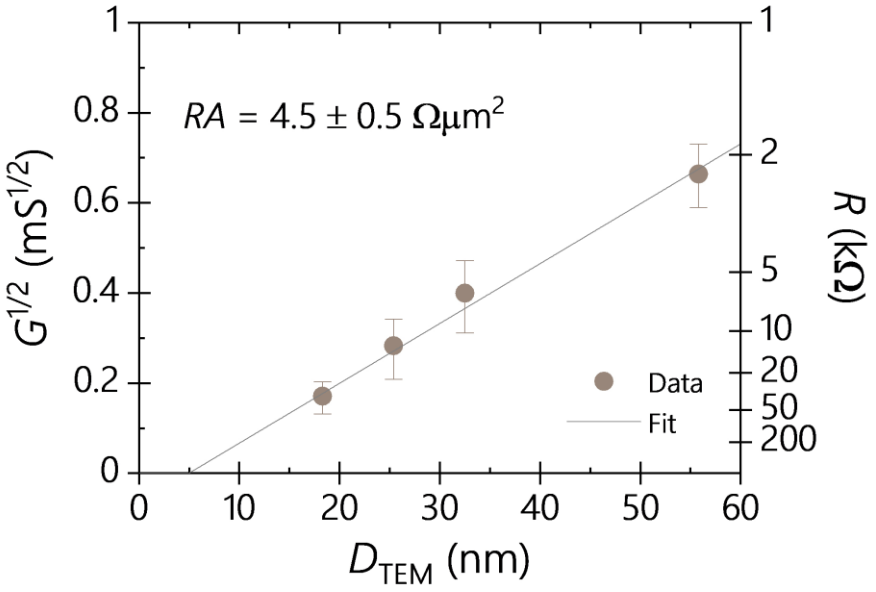

Supplementary Figure 4 | Determination of resistance-area product *RA*. The square root of the MTJ conductance G as a function of MgO diameter D_{TEM} determined from TEM observation. The solid line is a fitting according to Supplementary Equation 6. The right axis shows the correspondent resistance R.

Supplementary Note 4. Switching probability measurement using pulse magnetic field

In order to quantify the thermal stability factor $\varDelta$ of nano MTJs, switching probability measurement is carried out using pulse magnetic field with its duration τ of 1 s. The probability of magnetization reversal based on the Stoner-Wohlfarth model under an application of magnetic field is described as[6]

$$P_{\mathrm{P(AP)}} = 1 - \exp\left[-\frac{\tau}{\tau_0}\exp\left\{-\varDelta\left(1 \mp \frac{H - H_{\mathrm{shift}}}{H_{\mathrm{K}}^{\mathrm{eff}}}\right)^2\right\}\right], \tag{7}$$

where subscript P(AP) designates the magnetization switching from parallel (anti-parallel) to anti-parallel (parallel). τ_0 is the inverse of attempt frequency assumed to be 1 ns, H_{shift} is the shift field and $H_{\mathrm{K}}^{\mathrm{eff}}$ is the effective magnetic anisotropy field. Supplementary Figures 5a and 5b show the R-H loop and the switching probability as a function of the amplitude of magnetic-field pulse for

an MTJ with $D = 7.3 \pm 0.4$ nm. The result of the switching probability is fitted by Supplementary Equation 7 with Δ, H_K^{eff} and H_{shift} as fitting parameters, leading to $\Delta = 91 \pm 2$, $\mu_0 H_K^{eff} = 207 \pm 2$ mT and $\mu_0 H_{shift} = 10.2 \pm 0.1$ mT.

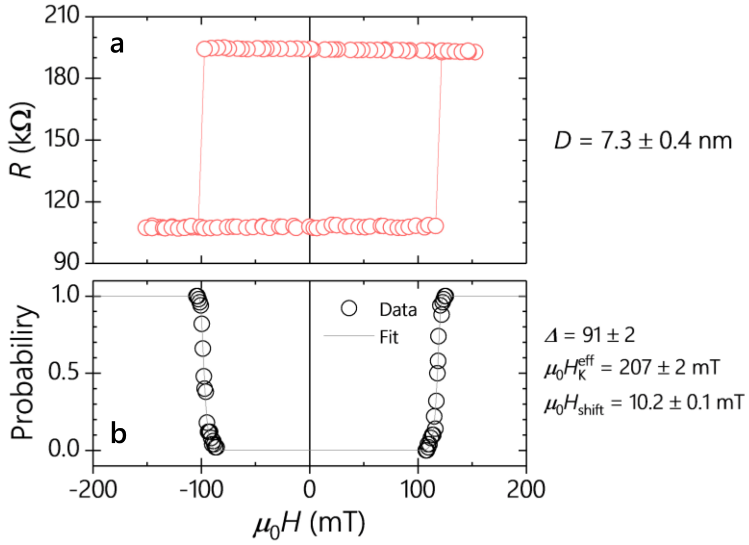

Supplementary Figure 5 | *R*-*H* loop and determination of *Δ*. a, an *R*-*H* loop measured for an MTJ with *D* = 7.3 nm. **b**, Switching probability as a function of 1 s-long pulse magnetic field and the fitting according to Supplementary Equation 7.

Supplementary References

1 Osborn, J. A. Demagnetizing Factors of the General Ellipsoid. *Phys. Rev.* **67**, 351-357 (1945).

2 Aharoni, A. *Introduction to the Theory of Ferromagnetism*. (Oxford University Press Inc., 1996).

3 Watanabe, K. *et al.* Annealing temperature dependence of magnetic properties of CoFeB/MgO stacks on different buffer layers. *Jpn. J. Appl. Phys.* **56**, 0802B2 (2017).

4 Okada, A. *et al.* Magnetization dynamics and its scattering mechanism in thin CoFeB films with interfacial anisotropy. *Proc. Natl. Acad. Sci. USA* **114**, 3815-3820 (2017).

5 Gajek, M. *et al.* Spin torque switching of 20 nm magnetic tunnel junctions with perpendicular anisotropy. *Appl. Phys. Lett.* **100**, 132408 (2012).

6 Li, Z. & Zhang, S. Thermally assisted magnetization reversal in the presence of a spin-transfer torque. *Phys. Rev. B* **69**, 134416 (2004).
